# Supplementary material for: Phages ZC01 and ZC03 require type-IV pilus for Pseudomonas aeruginosa infection and have a potential for therapeutic applications
Source: Microbiol Spectr. 2024 Oct 29;12(12):e01527-24. doi: 10.1128/spectrum.01527-24 (PMC11619397; doi:10.1128/spectrum.01527-24)
Supplement: Fig. S1 — One-step growth curve for phage ZC01. [file spectrum.01527-24-s0001.pdf]

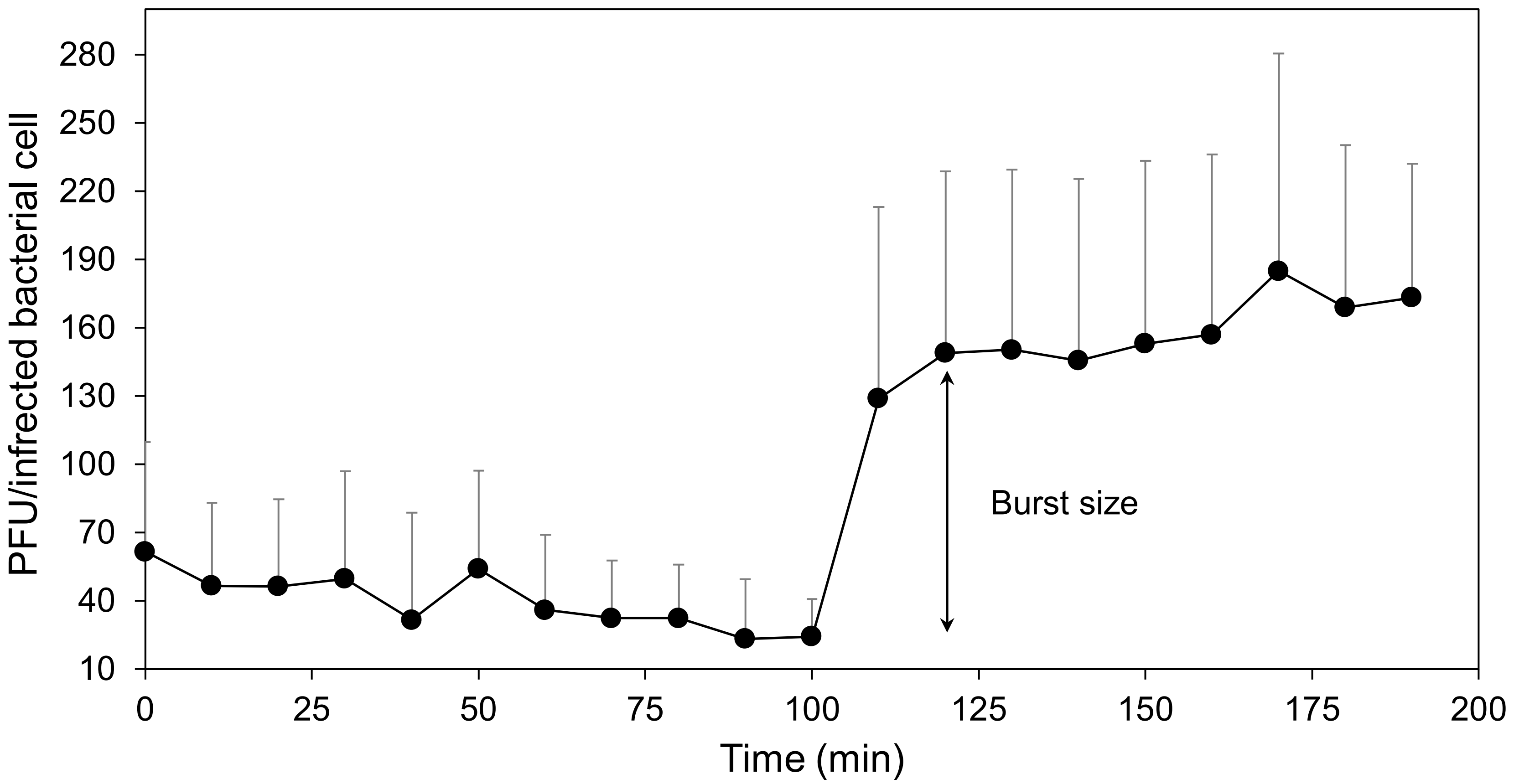

Figure S1. One-step growth curve for phage ZC01 showing a latent period of ~100min. A plateau is reached at ~120min and used to calculate the burst size of 87 phage particles per infected cell. Bars indicate mean ± standard deviation of three independent experiments. The curve was performed using *P. aeruginosa* PA14 as host.
