# Supplementary material for: Phages ZC01 and ZC03 require type-IV pilus for Pseudomonas aeruginosa infection and have a potential for therapeutic applications
Source: Microbiol Spectr. 2024 Oct 29;12(12):e01527-24. doi: 10.1128/spectrum.01527-24 (PMC11619397; doi:10.1128/spectrum.01527-24)
Supplement: Fig. S2 — Time-killing curves of P. aeruginosa PA14 exposed to phages ZC01 and ZC03. [file spectrum.01527-24-s0002.pdf]

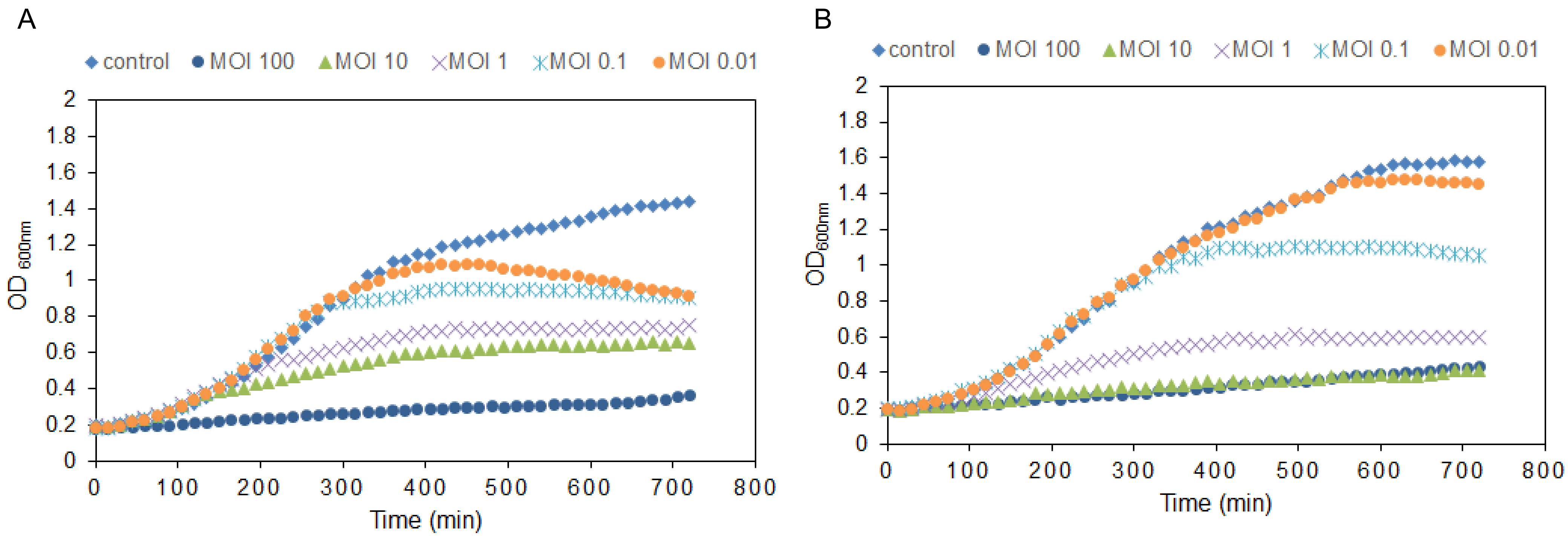

Figure S2. Time-killing curves of *P. aeruginosa* PA14 exposed to phages ZC01 and ZC03. Lytic activity of ZC01 (A) and ZC03 (B) was accessed by mixing PA14 suspensions (1×10^6 CFU/mL) with phages at MOIs of 0.01, 0.1, 1, 10 and 100 and incubated at 37 °C for 720 min. Bacterial growth was followed by OD_{600nm} measurements every 15 min during the incubation. Assays were undertaken independently in duplicate with six technical replicates for each sample.
