# Supplementary material for: Phages ZC01 and ZC03 require type-IV pilus for Pseudomonas aeruginosa infection and have a potential for therapeutic applications
Source: Microbiol Spectr. 2024 Oct 29;12(12):e01527-24. doi: 10.1128/spectrum.01527-24 (PMC11619397; doi:10.1128/spectrum.01527-24)
Supplement: Fig. S3 — ZC01 and ZC03 phage particles subjected to SDS-PAGE. [file spectrum.01527-24-s0003.pdf]

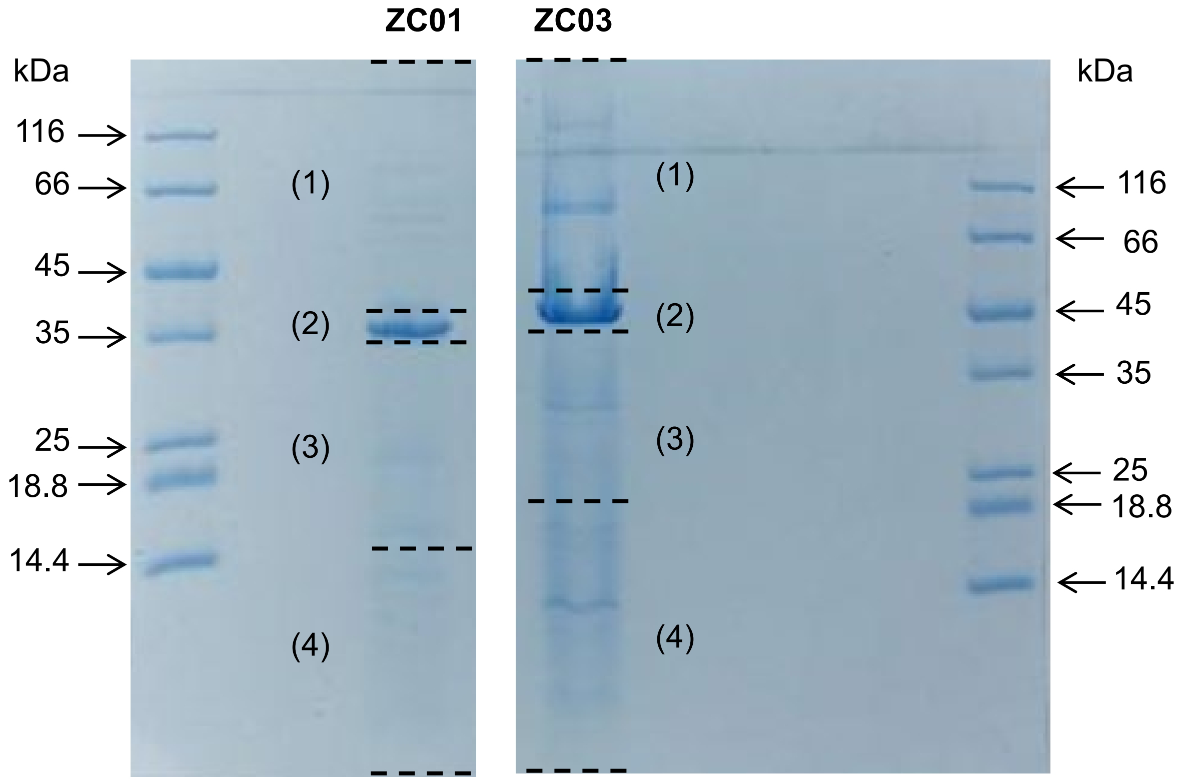

Figure S3. ZC01 and ZC03 phage particles (~100μg of total protein) were subjected to 15% SDS-PAGE. Polypeptides were visualized by staining with 0.1% Coomassie blue G250. After destaining, the gel regions were cut out (dashed lines), transferred to microtubes for in-gel digestion and mass spectrometry-based proteomics. The molecular masses of marker proteins in kDa are indicated.
